# Development of a PNA Probe for Fluorescence *In Situ* Hybridization Detection of *Prorocentrum donghaiense*

**DOI:** 10.1371/journal.pone.0025527

**Published:** 2011-10-14

**Authors:** Guofu Chen, Chunyu Zhang, Baoyu Zhang, Guangce Wang, Douding Lu, Zhong Xu, Peishen Yan

**Affiliations:** 1 State Key Laboratory of Urban Water Resource and Environment, Harbin Institute of Technology, Harbin, China; 2 Tianjin Key Laboratory of Marine Resources and Chemistry, Tianjin University of Science and Technology, Tianjin, China; 3 Institute of Oceanology, Chinese Academy of Sciences, Qingdao, China; 4 The First Institute of Oceanography, SOA, Qingdao, China; 5 The Second Institute of Oceanography, SOA, Hangzhou, China; University of Melbourne, Australia

## Abstract

*Prorocentrum donghaiense* is a common but dominant harmful algal bloom (HAB) species, which is widely distributed along the China Sea coast. Development of methods for rapid and precise identification and quantification is prerequisite for early-stage warning and monitoring of blooms due to *P. donghaiense*. In this study, sequences representing the partial large subunit rDNA (D1–D2), small subunit rDNA and internal transcribed spacer region (ITS-1, 5.8S rDNA and ITS-2) of *P. donghaiense* were firstly obtained, and then seven candidate DNA probes were designed for performing fluorescence *in situ* hybridization (FISH) tests on *P. donghaiense*. Based on the fluorescent intensity of *P. donghaiense* cells labeled by the DNA probes, the probe DP0443A displayed the best hybridization performance. Therefore, a PNA probe (PP0443A) analogous to DP0443A was used in the further study. The cells labeled with the PNA probe displayed more intensive green fluorescence than that labeled with its DNA analog. The PNA probe was used to hybridize with thirteen microalgae belonging to five families, i.e., Dinophyceae, Prymnesiophyceae, Raphidophyceae, Chlorophyceae and Bacillariophyceae, and showed no visible cross-reaction. Finally, FISH with the probes PP0443A and DP0443A and light microscopy (LM) analysis aiming at enumerating *P. donghaiense* cells were performed on the field samples. Statistical comparisons of the cell densities (cells/L) of *P. donghaiense* in the natural samples determined by FISH and LM were performed using one-way ANOVA and Duncan's multiple comparisons of the means. The *P. donghaiense* cell densities determined by LM and the PNA probe are remarkably higher than (*p*<0.05) that determined by the DNA probe, while no significant difference is observed between LM and the PNA probe. All results suggest that the PNA probe is more sensitive that its DNA analog, and therefore is promising for the monitoring of harmful algal blooms of *P. donghaiense* in the future.

## Introduction

The occurrence of harmful algal blooms (HABs) reportedly has been increasingly on a global scale, which is associated with a series of economic and environmental problems [1]. To warn of the occurrence of HABs and avoid the loss due to them, strict monitoring of the causative algae is necessary. Therefore, precise detection methods should be developed to facilitate the identification and quantification of harmful algae.

*Prorocentrum donghaiense*, which belongs to Dinophyta, Dinophyceae, Prorocentrophycidae and Prorocentrales, is a common *Prorocentrum* species widely distributed along the China coast. Meanwhile, this species has always been one of the most dominant HABs species in the East China Sea since 2000 [2], [3]. It has also been reported that blooms of the same species have occurred in Japan, South Korea and Turkey. In China several major blooms of over 1000 km^2^ have occurred in the last decade causing significant local concern [4]. Considering its negative impact on the marine ecosystem, aquaculture and public health, it is essential for precise identification and quantification in the phytoplankton research and to provide important data for water quality assessment and early warning of the hazards of *P. donghaiense* to fisheries and aquaculture.

Unfortunately, correct identification and enumeration of *P. donghaiense* is not trivial. The cells are smallish, with a length of 16–22 µm and width of 9.5–14 µm, and are fragile and cell morphology often changes under different water conditions [3]. This species has not been recognized for a long time until it was first reported and established by Lu and Goebel [5] in 2001. Even after the establishment of *P. donghaiense*, it has also been confused with another related species *P. dentatum* [3], [6], [7]. Specially, the taxonomy of *P. donghaiense* has been very recently discussed in Percopo et al [8]. This paper has commented the similarity of *P. donghaiense* and *P. maximum*, indicating a potential synonymy of the two species, which is however still not resolved due to the lack of taxonomical information on *P. maximum*. One clear implication is that much experience is required to identify and enumerate *P. donghaiense* by light and electron microscopy using morphological characters known to be present in both cultured and wild samples. Things become more complicated when *P. donghaiense* is only a minor component of plankonic assemblages, or when trying to distinguish between morphologically similar species or strains, such as *P. dentatum*, *P. minimum* and *P. micans*. Moreover, the traditional methods relying on microscopical examination is laborious, tedious and time-consuming, especially when large numbers of samples are to be analyzed. For the above reasons it is necessary to develop a simple, rapid, and effective identification and quantification method for this species.

In previous studies, biochemical, immunological and molecular techniques have been introduced to facilitate identification and enumeration of phytoplankton [8]. Among these, molecular methods are the most favored, because they aim for nucleic acid in cells, which is relatively invariable compared with other target molecules. Lots of techniques, including fluorescence *in situ* hybridization (FISH) [8], [9], real-time PCR [10], [11], sandwich hybridization assay (SHA) [12], [13], loop-mediated isothermal amplification [14], nuclease-protection-assay/sandwich hybridization (NPA-SH) [15] and nucleic acid sequence-based amplification (NASBA) [16] have been reported. However, few efforts were made on *P. donghaiense*. Polyclonal antibodies targeting cell surface antigens of *P. donghaiense* were firstly developed by Wang et al. [17]. Despite that this method could distinguish *P. donghaiense* from other unrelated species, the antiserum against *P. donghaiense* showed weak cross-reactions with the closely related species. Another problem is that the detection reliability needs to be further tested, since the cell surface tends to change with water conditions. Moreover, the serum preparation is comparatively complicated and troublesome. Recently, Chen et al. [2] established an assay for *P. donghaiense* with NPA-SH. However, this method requires the quantitative extraction of high quality RNA, which is more difficult for *Prorocentrum* with hard thecae than for fragile and naked species (e.g. *Heterosigma akashiwo*) [18], [19]. Specially, uniform extraction of RNA from a diverse range of organisms is necessary for environmental monitoring. These suggest that NPA-SH may be not promising.

FISH is a technique for *in situ* detection of unicellular microbial organisms [20], [21], which has been widely used for detection and enumeration of a few harmful algae. Despite that FISH is a promising method, the observation of fluorescent cells in field samples is sometimes problematic for some species, because the fluorescence of cells labeled with DNA probes may be rather weak. *P. donghaiense* is unfortunately a member of these species according to the findings from Zhang *et al.* [22]. In their study, they firstly explored the utility of an rDNA-targeted oligonucleotide probe to detect *P. donghaiense* cells using FISH, but fail to obtain labeled cells of intensive fluorescence.

Peptide nucleic acid (PNA) probes may be a good alternative to DNA probes, which are widely used in the current FISH analysis. PNA probes are synthetic DNA mimics, with sugar phosphate backbone of DNA helix replaced by uncharged structurally homomorphous pseudopeptide backbone [23]–[25]. PNA probes with synthetic backbone are characteristic of more rapid and stronger binding capability [26], [27], much higher specificity [28], hybridization efficiency [29] and hybridization stability [23], [28] than their DNA analogs. To date, PNA probes targeting rRNA have only been sparsely applied in phytoplankton studies, including *in situ* probing [26] and rRNA quantification [30] of *Prochlorococcus and Synechococcus* cells, and a life cycle study of *Pfiesteria piscicida* [31]. Recently, PNA probes were also introduced to monitor harmful algae. A semi-automated SHA employing a PNA signal probe could enhance the detection level of *Alexandrium tamarense* [27]. Another PNA probe for the detection of the toxic dinoflagellate *Takayama pulchella* was also developed [9]. Generally, the few current studies demonstrate that PNA probes should be useful for monitoring harmful algae.

For reasons such as noted above, this study focused on the development of a PNA probe for *P. donghaiense*, and explored its potential application to detect target species in field samples. We firstly PCR amplified, cloned and sequenced the partial large subunit rDNA D1–D2 (LSU D1–D2), small subunit rDNA (SSU rDNA), and internal transcribed spacers region (ITS-1, 5.8S rDNA and ITS-2), and then designed candidate probes to screen the best probe for FISH detection of *P. donghaiense* by laboratory and field tests.

## Results and Discussion

### Probes design

The final aim of this study is to develop a PNA probe for FISH detection of *P. donghaiense*. Screening an optimal probe among few candidate probes is crucial for this. Direct PNA probe screening must be costly, since the current price of a PNA probe is more than 10 times higher than that of its DNA analog. Therefore, we obtained the optimal probe of best hybridization performance by testing a few DNA probes, and then used its PNA analog for the further study.

So far, the probes targeting rRNA have been widely used for FISH detection of several harmful algae [12], [20], with less work done to develop rDNA-targeted probes [8], [32]. In this study, a wide range of probes were screened from the LSU D1–D2, SSU rDNA, and ITS sequences, among which both the LSU D1–D2 and SSU were used for rRNA targeting probes, while the ITS for rDNA targeting probes design. BLAST search and alignment analysis showed that different stains of *P. donghaiense* have identical nucleic acid sequences of LSU D1–D2, SSU rDNA and ITS (data not shown), implying that they are conservative and competent for probe design for different strains of the species. However, they display comparatively different variability within *Prorocentrum*. Among them, LSU D1–D2 shows higher variable degree, whereas SSU rDNA and ITS are relatively conservative to be difficult to search for specific regions. Remarkably, the conservation of the ITS sequence of *P. donghaiense* is out of expectation, since more findings demonstrate that many species usually have more variable ITS than their LSU and SSU [33], [34] due to the less evolutional pressure and relatively rapid divergence rates [35]. Finally, a total of 9 DNA probes, including 4 targeting LSU rRNA (DP0587A22, DP0602A23, DP0512A19 and DP0443A19), 1 targeting SSU rRNA (DP1704A23), 2 targeting ITS rDNA (DP0159A25 and DP0498A21), and 2 control probes (DU0512A18 and DU0499S18) [36]–[38], were introduced for further probes screening, as shown in Table 1.

**Table 1:** Summary of probes introduced into FISH analysis.

Probes^a^	Sequences (5′– 3′)	Target nucleic acid	Aligned position
DNA-UniC-0512-A-18	GWATTACCGCGGCKGCTG	cytoplasmic SSU RNA	512–529
DNA-UniR-0499-S-18	CAGCMGCCGCGGUAAUWC		
DNA -Pdon-0587(*P. donghaiense*)-A-22	TTTGGCACCTTGGAGATCTCGG	cytoplasmic LSU RNA	587–608
DNA -Pdon-0602(*P. donghaiense*)-A-23	ATCTCGGCTTGGCCTGCCACAGT	cytoplasmic LSU RNA	602–624
DNA-Pdon-0512(*P. donghaiense*)-A-19	CTTGTCTTCGGGTGAGTGA	cytoplasmic LSU RNA	512–530
DNA -Pdon-0443(*P. donghaiense*)-A-19	TCCTGATCGTCTCCTGCCT	cytoplasmic LSU RNA	443–461
DNA -Pdon-1704(*P. donghaiense*)-A-23	GGACCTGGACGAACGCCTTTCAA	cytoplasmic SSU RNA	1704–1726
DNA-Pdon-0159(*P. donghaiense*)-A-25	CCACTCAGAACAAATTGGAACATAC	nuclear ITS DNA	159–183
DNA-Pdon-0498(*P. donghaiense*)-A-21	GCCCGACAACAAGACAACAGA	nuclear ITS DNA	498–518
PNA -Pdon-0443(*P. donghaiense*)-A-19	TCCTGATCGTCTCCTGCCT	cytoplasmic LSU RNA	443–461

a Probe names follow the nomenclature outlined by Wheeler Alm et al. [51], with little revision. The first four letters stand for the kind of probe; for example, PNA stands for PNA probe. The second four-letter code is for the target of the probe. The next number is the 5′ position of the probe relative to either *Escherichia coli* or target organism (*P. donghaiense*). The next letter is for whether the probe is identical to the DNA sense or antisense strand. The last number is the length of the probe.

### Probes screening

**Figure 1:**
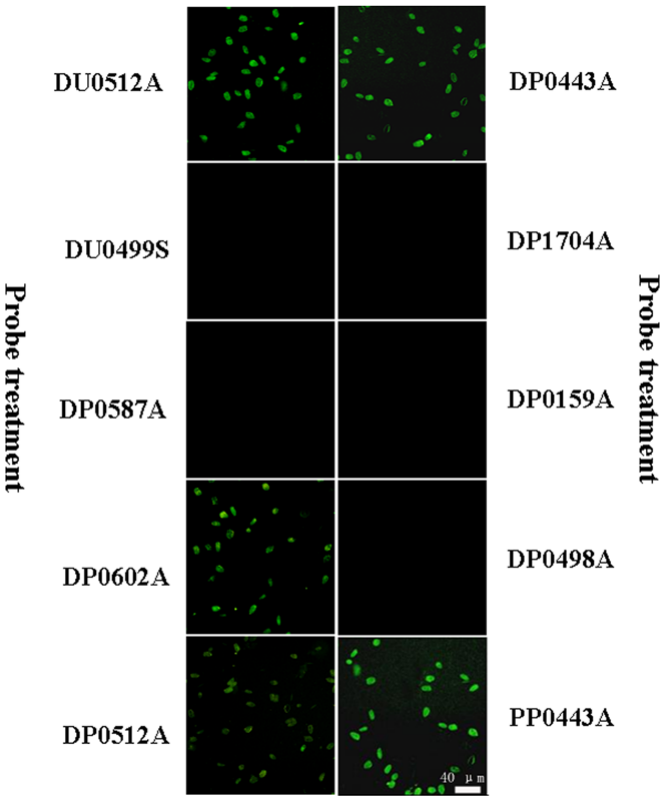
Representative micrographs of the FISH analyses showing sensitivity of probes to *Prorocentrum donghaiense*.

The results of FISH using all the DNA probes are summarized in Table 2 and Fig. 1. *P. donghaiense* could not be labeled by the probes targeting both SSU rRNA (DP1704A23) and ITS rDNA (DP0159A25, DP0498A21). The complex second structure of rRNA may preclude its hybridization with DP1704A23, since rRNA expression in cells is often thought to be at a high level. Except for certain species [8], rDNA is generally thought to be unsuitable for probe targeting, because the cells labeled with rDNA targeting probe tend to display weak fluorescence [32], which disturb their differentiation from other species in natural samples [39]. Things seem to get worse for *P. donghaiense*, since the cells marked by both DP0159A25 and DP0498A21 did not display any visible fluorescence under epifluorescence microscopy. The possible reason for this is that the copies of ITS rDNA within genomic DNA of *P. donghaiense* are at least less than *A. catenella* [32], *A. tarmarense* [32] and *H. akashiwo* [8]. Therefore, *P. donghaiense* cells could not provide enough biding molecules for the rDNA targeting probe, and the hybridized cells with less fluorescein labeled probe naturally give out weak and even invisible fluorescence, as shown in this study.

**Table 2:** Sensitivity of probes to *Prorocentrum donghaiense* determined by the FISH assays^a^.

Probes	DU0512A	DU0499S	DP0587A	DP0602A	DP0512A	DP0443A	DP1704A	DP0159A	DP0498A	PP0443A
Sensitivity	+++	−	−	++	+	+++	−	−	−	++++

a Cells with signal intensity similar to the positive control were scored as “+++”; signal intensity equivalent to the negative control was scored as “−”; signal intensities clearly above the negative but below the positive control were scored as “++” or “+”, depending on the brightness relative to the positive and negative probes; signal intensity above the positive control was scored as “++++”.

**Figure 2:**
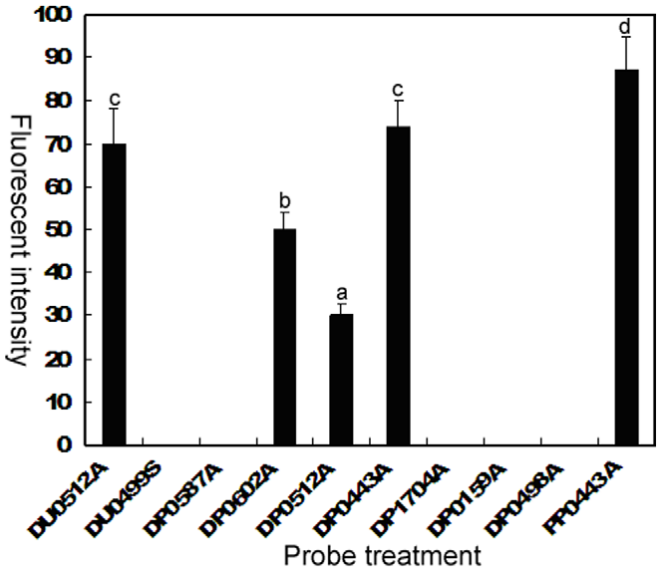
Fluorescent intensity of cells labeled with different probes. Values are mean ± SE (n = 20). Different letters indicate significant differences (*p*<0.05) determined by one-way ANOVA and Duncan's multiple comparisons.

The effect of the secondary structure of the LSU rRNA on the accessibility of probes to the target sites has been shown in previous studies [40]–[42]. Again, our findings reconfirm this. The four rRNA-targeted probes with even slight alternation in the 5′ portion of the sequence displayed different performance (Table 2 and Fig. 1). Among them, only DP0443A labeled *P. donghaiense* cells with fluorescent intensity equivalent to the positive control probe (DU0512A18), while *P. donghaiense* cells marked by DP0587A did not show any fluorescence. The cells labeled with DP0602A and DP0512A displayed more or less intensive fluorescence compared with the positive control probe labeling cells, respectively. The further quantification analyses of fluorescent intensity of cells labeled with different probes were shown in Fig. 2. Apparently, the fluorescent intensity of DP0443A labeling cells were significantly more intensive (*p*<0.05) than that of the cells marked by other LSU rRNA-targeted probes.

Based on these findings, DP0443A could be considered as the best among these designed DNA probes. Consequently, we synthesized a PNA probe (PP0443A) with same nucleotide sequence to DP0443A and utilized it to hybridize with *P. donghaiense*. As expected, the PNA probe PP0443A labeled *P. donghaiense* cells with more intensive fluorescence than the positive control and DP0443A (Fig. 1). Moreover, the difference in fluorescent intensity between them was significant (*p*<0.05) (Fig. 2). Thus, we gain the ideal PNA probe for FISH detection of *P. donghaiense*.

### Specificity of the PNA probe

The specificity of the PNA probe (PP0443A) should be considered as a critical point for FISH detection. To achieve this, the probes were firstly designed based on the multiple sequence alignment involving the LSU D1–D2 sequences of *P. donghaiense* and all other *Procentrum* available in Genbank. Next, BLAST searches were performed on the designed probes, confirming that the sequences of probes could exclusively match with *P. donghaiense*. Finally, cross-reactivity of the screened probe against other microalgae was tested. The positive (DU0512A) and negative (DU0499S18) control treatments were included to define a range of labeling intensities possible for any given sample and thereby provided a reference from which to assess the reactivity of specific probe.

The FISH trials served as an intermediate step to determine whether a candidate probe could access its target sequence. Therefore, no attempt was made to optimize the whole cell hybridization conditions and the list of species used in the trials was also limited. The results of hybridization with all test species using the PNA probe and control probes are shown in Table 2. The positive probe could react with all test species, repeatedly giving bright and uniform label intensity for all species examined. Contrarily, the negative probe could not label any species, and the cells treated by negative probe appeared uniformly dark. In contrast, PP0443A reacted exclusively with *P. donghaiense*. Based on these, the specific PNA probe may be speculated to be useful for molecular identification of the target species in natural samples containing many different microalgae.

### Application of DNA and PNA probes to detect *P. donghaiense* in natural samples

**Figure 3:**
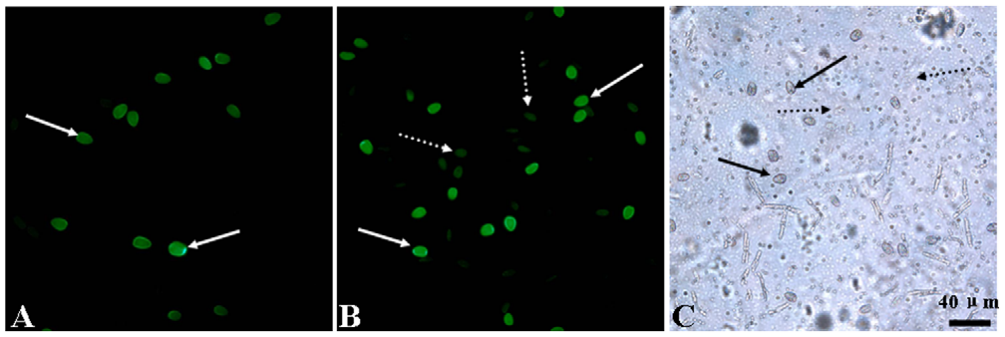
ISH analysis of natural sample. A: FISH with probe DP0443S; B: FISH with probe PP0443S; C: LM. Arrows denote normal *Prorocentrum donghaiense* cells, while dotted-line arrows denote probably dying or dead cells.

Both the DNA (DP0443A) and PNA (PP0443A) probes were used to analyze twelve natural samples from different stations located in the East China Sea. The representative micrographs of FISH analysis are shown in Fig. 3. Some dying or dead target cells, deduced from their blurry contours with weaker color compared with the surounding living cells under light microscope (LM), were observed to be included in the field samples (Fig. 3 C). Both the DNA and PNA probes could enter the algal cells easily and bound strongly with the target species, rendering the target cells green (Fig. 3 A, B). However, the PNA labeled cells were expected to give stronger fluorescence on average than the DNA probe labeled cells (Fig. 3 A, B). The reason for this is that the PNA probe has much stronger binding capability [26], [27] and higher hybridization efficiency [29] than its DNA anolog. This also explains why the dying or dead cells could well be stained by the PNA probe, but scarcely stained by the DNA probe (Fig. 3 B, C). Moreover, the hybridizations with both probes are specific, since only the *P. donghaiense* cells were labeled in the field samples, without non-specific binding to other algal species (Fig. 3).

**Figure 4:**
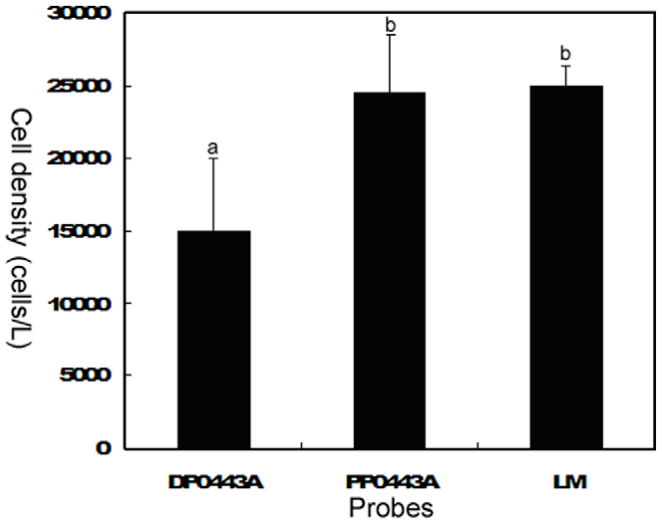
Cell density (cells/L) of *Prorocentrum donghaiense* in natural samples determined by FISH with DNA (DP0443A) and PNA (PP0443A) probes and light microscopy (LM). Values are mean ± SE (n = 12). Different letters indicate significant differences (*p*<0.05) determined by one-way ANOVA and Duncan's multiple comparisons.

All the natural samples were used for direct enumeration by LM and indirect enumeration after FISH treatments with both the DNA and PNA probes. The results showed that the *P. donghaiense* cell densities determined by LM and the PNA probe were remarkably higher than that determined by the DNA probe (*p*<0.05) (Fig. 4). No significant difference was observed between the cell densities determined by LM and PNA probe (Fig. 4). Whether the dying or dead cells were stained or not due to the sensitivity may be one of the most possible reasons for the difference in cell densities between the DNA and PNA probes. Obviously, the PNA probe is more competent for target cell enumeration than the DNA probe. These also indicate that the PNA probe and the hybridization protocol are effective for the detection of *P. donghaiense* in the field samples.

Many factors are speculated to influence efficiency and detection sensitivity of a molecular probe, such as sample treatment methods, autofluorescence of chlorophyll, and physiological station of target cells. Firstly, several necessary steps are usually taken to deal with the samples prior to observing fluorescent labeled target cells under the epifluorescence microscope. Lots of target cells are likely to be lost in the sample treatment steps, such as repeated centrifugation, pipetting, and washing in the earlier studies [32], [39]. This is specifically not fit for the natural samples in which the target species is a minor component. However, the subsequent filtration methods for the capture of target cells in the field samples [9], [20], as being adopted in this study, have already overcome this problem, avoiding the loss of even single cell. Secondly, red autofluorescence from abundant chlorophylls in algal cells could interfere with observation of the green fluorescence of target cells, which would possibly result in an underestimation of target cells. Therefore, an additional decolorization is likely a prerequisite prior to FISH analysis. This is sometimes true for the cells fixed by paraformaldehyde, which need a further ethanol or acetone treatment to reduce autofluorescence [43], [44]. However, the cells treated with the more widely used saline ethanol fixative are often competent for direct FISH analysis, without additional decolorization, because ethanol in the fixative could well destruct the chlorophylls. Some harmful algae, such as *P. micans* and *Karenia* spp. are exceptional (data not shown). When performing FISH analysis on them, the further methanol treatment to remove intensive red autofluorescence is necessary. Fortunately, the autofluorescence of *P. donghaiense* cells fixed by ethanol-based fixative was entirely removed. Thirdly and finally, varying rRNA content at different stage of target cells has been speculated to cause the detection efficiency variation [39], [42], [45]. However, the previous studies have shown that the variability of rRNA content does not influence the practical application of rRNA-targeted DNA probe, since the defection efficiency is relatively stable regardless of a little change in the fluorescence signal within a growth cycle [9], [39], [42]. Despite that the relationship between the growth stage and the detection efficiency is not investigated in this study, it is surprising to find the PNA probe could but the DNA probe could not labeled the dying or dead cells (Fig. 3 B, C), in which rRNA may mostly be decomposed. This also suggests that algal physiology could cause variation in detection efficiency of *P. donghaiense* for DNA probe due to varying rRNA content. Given the long time often taken to ship samples to the laboratory rRNA in cells may gradually decompose which should lead to reduced fluorescent intensity of labeled cells [20]. However, the more sensitive PNA probe will work well despite of less rRNA content. Therefore, it could be inferred that the PNA probe should be more suitable than its DNA analog for FISH analysis of field samples preserved for a long time.

In summary, the hybridization protocol adopted in this study is competent, and the PNA probe is more sensitive that its DNA analog, and therefore is promising for the monitoring of *P. donghaiense* in the natural samples in the future.

## Materials and Methods

### Algal cultures

Clonal *P. donghaiense* and other microalgae employed in this study were shown in Table 3. All the cultures were established by pipeting single cells or chains of cells, sequentially through droplets of sterile seawater. Cultures were grown at 20–22°C in Guillard's f/2 medium [46] on a 12∶12-h light∶dark cycle with light provided by cool white fluorescent tubes at a photon flux density of 50–100 µmol m^−2^ s^−1^. Silicate (110 µM) was added to the f/2 medium to support the growth of *Skeletonema* (used for probe cross-reactivity testing). All cultures were maintained in 250 ml flasks containing 100 ml f/2 (+Si) medium.

**Table 3:** List of species investigated in this study.

Species	Geographic origin
*Prorocentrum donghaiense*	East China Sea, West Pacific Ocean
*Prorocentrum minimum*	East China Sea, West Pacific Ocean
*Prorocentrum micans*	East China Sea, Zhejiang, China
*Prorocentrum dentatum*	Daya Bay, Guangdong, China
*Alexandrium tamarense*	East China Sea, West Pacific Ocean
*Karenia* sp1	Wenzhou, East China Sea, West Pacific Ocean
*Karenia* sp2	Hangzhou, East China Sea, West Pacific Ocean
*Gymnodinium* sp.	Jiaozhou Bay, Yellow Sea, West Pacific Ocean
*Phaeocystis globosa*	Daya Bay, Guangdong, China
*Heterosigma akashiwo*	Jiaozhou Bay, Yellow Sea, West Pacific Ocean
*Platy-monas cordiformis*	Bohai Sea Bay, West Pacific Ocean
*Skeletonema tropicum*	Qingdao Fishery, Yellow Sea, West Pacific Ocean
*Skeletonema dohrnii*	Jiaozhou Bay, Yellow Sea, West Pacific Ocean
*Skeletonema costatum*	Xiamen, Taiwan Strait, West Pacific Ocean

### DNA extraction, PCR amplification, cloning and sequencing

Total genomic DNA was isolated according to the protocol described previously by Chen et al. [8]. The LSU D1–D2, SSU and ITS sequences were specifically amplified by PCR with the universal primer pairs, D1 (5′-ACCCGCTGAATTTAAGCATA-3′)/D2(5′-CCTTGGTCCGTCTTTCAAGA-3′) [47], 6S1N (5′-TCCTGCCAGTAGTCATATGC-3′)/16S2N (5′-TGATCCT TCT/CGCAGGTTCAC-3′) [48], and TW81(5′-GGGATCCGTTTCCGTAGGTGAACCTG C-3′)/AB28(5′-GGGATCCATATGCTTAAGTTCAGCGGGT-3′) [49], [50] using a DNA Thermal Cycler (Takara, Dalian, China), respectively. The amplification conditions were as follows: denaturing at 94°C for 4 min, followed by 29 cycles of 94°C 1 min, 50°C 50 s, 72°C 50 s, and a final extension at 72°C for 7 min. Amplification products were purified and recycled using TIANquick Midi Purification Kit (TIANGEN Biotech Co., Ltd., Beijing, China) according to the manufacturer's instructions. Purified PCR products were ligated with pMD 18-T Vector (Sangon Biotech Co., Ltd, Shanghai, China) and transformed into competent *Escherichia coli* DH-5α (Sangon Biotech Co., Ltd, Shanghai, China). The positive colonies containing the objective DNA fragments were identified by colony PCR and then sequenced using Vector primer M13 as sequencing primer. Sequencing was performed in Sangon (Shanghai) Biotech Co., Ltd. The obtained sequences were submitted to GenBank, acquiring the accession numbers of DQ336340 (LSU D1–D2), AY465116 (ITS), and DQ336054 (SSU).

### DNA alignment and probe design

The obtained LSU D1–D2, SSU and ITS sequences were used for BLAST search, respectively, and the corresponding sequences of all *P. donghaiense* strains and *Prorocentrum* spp. deposited in GenBank were downloaded. All sequences of *Prorocentrum* used in this study were shown in Table 4. Three independent alignments containing the LSU D1–D2, SSU and ITS sequences, respectively, were conducted using computer software BioEdit for visually searching for specific regions for *P. donghaiense*. Oligonucleotide probes targeting the SSU, ITS and LSU were designed with the help of Premier Primer 6.0, respectively. The candidate probes were then refined with the aid of Oligo 6.0, excluding unsuitable probes mainly according to the potential problems associated with secondary structure and homer/dimer formation. The probes were screened with BLAST to examine their specificity against a wide range of organisms. Both the DNA (Invitrogen Biotechnology Co., Ltd., Shanghai, China) and PNA (Paide Biotechnology, Chengdu, China) probes were synthesized commercially with fluorescein isothionate (FITC) attached to the 5′ end. The probes received in a lyophilized form were dissolved in 0.1 M Tris-HCl (pH 7.5) to a final concentration of 100 µM, and aliquots were stored at −20°C in the dark. The probes are named following a changed nomenclature firstly outlined by Wheeler Alm *et al.* [51]. Using the probe ‘DNA-Pdon-0587-A-22’ as an example, the first three letters stand for the kind of the probe. The second four-letter code is for the species targeted. The next number is the 5′ position of the probe relative to either *Escherichia coli* or target organism (*P. donghaiense*). The next letter is for whether the probe is identical to the DNA sense (S) or antisense (A) strand. The last number is the length of the probe. All probes used in this study are listed in Table 1. In the rest of the table, figures and text, the probe name is shortened for brevity: for example, DNA-Pdon-0587-A-22 becomes DP0587A.

**Table 4:** List of *Prorocentrum* introduced into alignment for design of probes, with GenBank accession numbers of their LSU rDNA, ITS, and SSU rDNA sequences.

Species	GenBank accession number(LSU)	GenBank accession number(ITS)	GenBank accession number(SSU)
*Prorocentrum donghaiense*	DQ336340, EU586259, AY863007, AY833516, AY822610	DQ336340, AY465116	DQ336054, AY803743, AJ841810, AY551272
*Prorocentrum minimum*	EU780639	DQ662403	AY803741, AY803740
*Prorocentrum micans*	EU780638	EU927531	AY803739
*Prorocentrum dentatum*	FJ823581	FJ823581	DQ336057, AY803742
*Prorocentrum balticum*	AF042816	EU927547	
*Prorocentrum rostratum*	EU244471	EU244471	
*Prorocentrum rhathymum*	EU165279	EU244466	EU287487
*Prorocentrum triestinum*	AF042815	EU927551	DQ004734
*Prorocentrum mexicanum*	DQ336183	AY886763	EU287485
*Prorocentrum lima*		FJ823582	
*Prorocentrum cassubicum*		EU244475	
*Prorocentrum compressum*		EU927558	
*Prorocentrum gracile*			AY443019
*Prorocentrum tsawwassenense*			EF657885

### Fluorescence *in situ* hybridization tests for optimal probe

Comparative study on the hybridization performance of candidate probes was performed to screen the best probe. Approximately 10 ml of mid-exponential culture was pipetted gently into a 50 ml centrifuge tube containing 30 ml of saline ethanol fixative [1.25 ml ddH_2_O, 3.75 ml 20×SET buffer (3.00 M NaCl, 20 mM EDTA, 0.40 M Tris HCl, pH 7.8) and 25 ml of 95% ethanol] [37]. The mixture was left to stand at room temperature for 5 min before gently mixing by inversion, allowed to stand for an additional hour, and then centrifuged at 6000 g for 2 min at 4°C. The supernatant was removed, and the fixed cells were washed twice in 5×SET hybridization buffer by centrifugation at 6000 g for 2 min at 4°C. About 1–1.5 ml of 5×SET hybridization buffer was added to re-suspend the precipitated cells. The pelleted cells were aliquoted to 1.5 ml Eppendorf tubes. After centrifugation at 6000 g for 2 min at 4°C, as much supernatant as possible was removed for each tube. Then, 200 µl of 5×SET hybridization buffers containing probes were added. For probes targeting nuclear ITS DNA, cells were incubated at 97°C for 3 min to denature genomic DNA and incubated on ice for 3 min prior to hybridization. The reaction tubes were incubated for 1 h at 45°C. After hybridization, the labeled cells were washed twice with 1×SET for 3 min at 50°C. The labeled cells were at once mounted on glass microscope slides with SlowFade Light antifade solution (Molecular Probes Inc., Eugene, OR, USA) for epifluorescence microscopic observation or stored at 4 or −20°C in the dark for future analysis.

### Image capture and quantification of fluorescent intensity of labeled cells

Both image capture and quantification of fluorescent intensity of labeled cells were carried out as described in Miller and Scholin [20]. Microscopic observations of cells were performed at 522 nm under an epifluorescence microscope (Nikon Eclipse E800, Tokyo, Japan) when stimulated with 494 nm wavelength and fluorescent micrographs of cells were taken with Nikon digital camera equipped with the microscope. For comparative study, the configuration of the microscope remained constant throughout all trials, and all images were captured using a manual exposure setting of 3-s integration with all other camera parameters at default settings. Images were analyzed using computer program Scion Image. The freehand selection tool was used to manually determine the mean pixel density of cells by defining labeled cells being analyzed. Twenty randomly selected cells were examined from each treatment and pixel density was averaged to provide a quantitative estimate of cell fluorescence intensity. The final cell fluorescence intensity was represented by the value of 255 subtracted by the mean pixel density of 20 cells.

### Cross reactivity test

The PNA analog (PP0443A) to the DNA probe (DP0443A) of the best hybridization performance was used to hybridize with thirteen microalgae cultured in our laboratory, including common HAB causative species, such as *P. minimum*, *P. micans*, *P. dentatum*, *Karenia* spp., *H. akashiwo*, *A. tarmarense*, *Phaeocystis globosa* and *Skeletonema* spp. (Table 3), following the already described FISH procedure for *P. donghaiense*.

### FISH and light microscopy (LM) analysis of field samples

Natural samples were collected from East China Sea, where the cell density of *P. donghaiense* bloom is commonly at 10^6^ cells/L [3]. The improved protocol for the filed material was summarized as follows. Briefly, 1.5 ml field sample was fixed for 30 min with 3.5 ml of saline ethanol solution, filtered using Whatman 25 mm diameter 0.2 µm pore size Nuclepore filter, and then rinsed twice with 1 ml of hybridization buffer (5×SET). Wrapped filters could be stored at 4°C for at least 4 weeks or processed immediately. Next, the filter was placed on a glass slide and 500 µl of probe (10 µM) (PP0443A or DP0443A) dissolved in 5×SET was added. The filter was hybridized in the dark for 1 h at 45°C, washed twice for 3 min at 50°C with 1 ml of pre-warmed washing buffer (1×SET) to remove excess probe. The labeled cells were examined and counted under an epifluorescence microscope. Also, the natural samples were used for direct enumeration by LM with haemacytometer. The morphological characters used to distinguish *P. donghaiense* from other taxa were as being described in Lu et al. [3], [7] and Lu and Goebel [5].

### Statistical analysis

Statistical analysis of fluorescent signal intensity of labeled cells was carried out using the software SPSS 13. One-way ANOVA and Duncan's multiple comparisons of the means were done to compare the data obtained.
